# Comparative study of electrochemical-based sensors and immunosensors in terms of advantageous features for detection of cancer biomarkers

**DOI:** 10.55730/1300-0527.3587

**Published:** 2023-08-09

**Authors:** Göksu ÖZÇELİKAY, Ahmet ÇETİNKAYA, S. İrem KAYA, Sibel A. ÖZKAN

**Affiliations:** 1 Department of Analytical Chemistry, Faculty of Pharmacy, Ankara University, Ankara, Turkiye; 2 Graduate School of Health Sciences, Ankara University, Ankara, Turkiye; 3 Department of Analytical Chemistry, Gulhane Faculty of Pharmacy, University of Health Sciences, Ankara, Turkiye

**Keywords:** Immunosensor, molecularly imprinted polymer, cancer, biomarker, biosensor

## Abstract

Cancer, becoming increasingly common globally, has a high mortality rate. Despite the much research on diagnosis and treatment methods, the benefits of technological developments, and newly developed sensor devices, cancer is still one of the leading causes of death worldwide. Early detection using powerful and noninvasive tools could be a future focus for prognosis and treatment follow-up. Therefore, electrochemical biosensors can be a strong choice for the detection of cancer biomarkers (such as alpha-fetoprotein, cytochrome c, prostate-specific antigen, myoglobin, carcinoembryonic antigen, alpha-fetoprotein, a cancer antigen, epidermal growth factor receptor, vascular endothelial growth factor, circulating tumor cell, and breast cancer antigen 1/2) due to their advantages such as high sensitivity, excellent selectivity, low cost, short analysis time, and simplicity. Furthermore, electrochemical biosensors are better suited for point-of-care applications due to their mass production and miniaturization ease. This review provides an overview of different electrochemical measurement techniques, bioreceptor surfaces, signal production and amplification, and the integration of electrochemical-modified sensors. Cancer biomarkers based on electrochemical biosensors were given in detail. In addition, studies with MIP-based sensors and immunosensors have been extensively discussed. Integrating electrochemical biosensors with cancer biomarkers was also emphasized as a new research trend. Finally, we provide an overview of current advances in measuring and analyzing cancer biomarkers using electrochemical biosensors and detail current challenges and future perspectives.

## 1. Introduction

Cancer is a disease that occurs because of uncontrolled proliferation and the product of cells in any organ or tissue of the body. Skin cancer, breast cancer, lung cancer, prostate cancer, gastric cancer, large intestine (colon) cancer, cervical (cervix) cancer, and lymph node tumors were generally observed worldwide. Cancer is one of the deadliest diseases. Therefore, early diagnosis is the most crucial factor in cancer treatment. While the cure rate of the disease diagnosed in the early period is between 80% and 90%, the cure rate of advanced cancer is around 40%–50%. Since early diagnosis is very important in cancer, biomarkers are needed [1].

Biomarkers are biomolecules that allow us to measure an organism’s abnormal or normal biological state. These biomolecules include nucleic acids, proteins such as enzymes and receptors, peptides, antibodies, and similar molecules. According to the World Health Organization, a biomarker is defined as “any substance, structure, and process that can be measured in the body, its products that influence and predict the occurrence of disease and its outcome.” Biomarkers differ according to the type of cancer. Evaluation of the diagnostic value of any cancer biomarker released from tumor cells is performed according to the sensitivity and specificity of this biomarker [2]. Exemplarily, the alpha-fetoprotein (AFP), carbohydrate antigen 125 (CA125), carbohydrate antigen 19-9 (CA 19-9), prostate-specific antigen (PSA), and carcinoembryonic antigen (CEA) biomarkers help to diagnose hepatocellular cancer, ovarian cancer, pancreas cancer, prostate cancer, and colon cancer respectively [3]. Biopsy analysis and tumor imaging are used for not only the diagnosis of cancer but also cancer biomarker monitoring through approaches such as enzyme-linked/radio/electrophoretic immunosorbent assay (ELISA), mass spectrometry, high-performance liquid chromatography (HPLC), and optical/electrochemical/thermal sensors.

Electrochemical sensors have several advantages: high sensitivity and selectivity, high chemical/mechanical stability, an easy preparation process, and miniaturization [4]. These properties of electrochemical sensors for target analytes show that they are a better approach for high-quality sensing applications than conventional device techniques and other sensor types.

**Figure 1:**
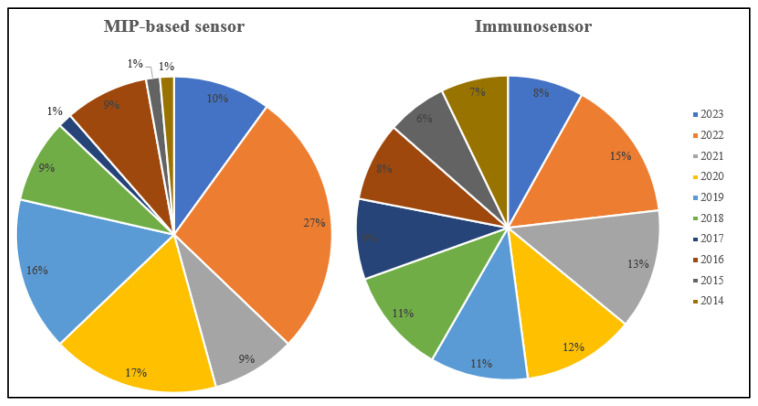
A comparative chart relevant to the articles of MIP-based sensors and immunosensors towards cancer biomarker detection from 2014 to 2023 (The date of access to Scopus database: 25.05.2023).

In particular, selectivity comes to the fore in developing electrochemical sensors to analyze biomarkers as target analytes. Different approaches can be used to provide and increase selectivity. Electrochemical immunosensors and MIP-based sensors are essential approaches in biomarker analysis with their specific recognition and high selectivity. These two types of electrochemical sensors are fabricated according to the recognition surface. The immobilization of antibodies as a recognition agent is a key point for immunosensor. The immunocomplex is formed with antibody-antigen interactions and integrated with an electrochemical transducer. Molecularly imprinted polymers (MIP) are cheap and easily prepared artificial materials suitable for molecular recognition with specific recognition sites specific to the target molecule. Molecularly imprinted polymers are synthetic systems formed by the monomers in the presence of the target molecule [5]. Electropolymerization, photopolymerization, and thermal polymerization are used to fabricate MIP-based sensors. After removal treatment, the cavities specific to the target molecule are formed in a polymeric matrix. The developed sensors can be applied to real samples. A comparative chart was given for the statistical evaluation of the articles from 2014 to 2023 relevant to the MIP-based sensors and immunosensors towards cancer biomarker detection. Considering the analysis studies of cancer biomarkers in the last 10 years, it is shown in Figure 1 that there are more immunosensor studies compared to MIP-based sensor studies.

The comparison of the principles, advantages, and disadvantages of MIP-based sensors and immunosensors analysis is given in Table 1. These biosensors are compared from different perspectives. There are no studies about the comparison of MIP-based biosensors and immunosensor in the literature.

This current review aims to be helpful to researchers by evaluating the popular cancer biomarkers, MIP-based biosensors/immunosensors applications of cancer biomarkers.

## 2. Cancer biomarkers

**Figure 2:**
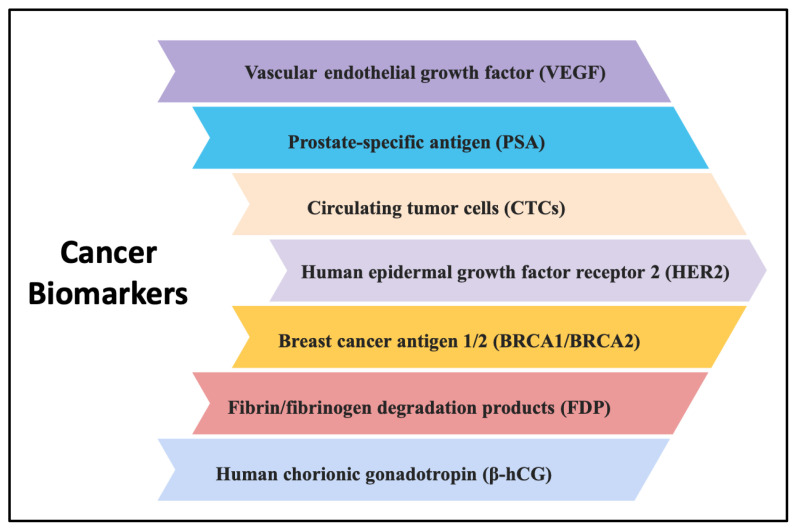
The list of popular cancer biomarkers.

Today, although there are many studies on cancer diagnosis and treatment approaches, the advantages of technological developments and newly developed sensor devices have made great progress, cancer is still one of the leading causes of death worldwide [6]. In this context, biomarkers have a very critical role and provide vital information about the early diagnosis of cancer, the progression of the disease and the risk of recurrence, and the monitoring of the efficacy of treatment by the determination of biomarkers from the biological fluids of individuals such as blood, saliva or urine [2]. The popular cancer biomarkers are listed in Figure 2.

Hence, cancer biomarkers are classified according to their structures, sources, and the type of cancer they indicate, and summarized in Table 2.

Each biomarker and its presence or increased/decreased levels can provide specific information about the cancer process. Additionally, biomarkers can be affected by changes in patient’s dietary habits and daily activities. Specific and selective identification of biomarkers is a challenging process, and cancer management includes other techniques such as optical imaging (ultrasound, MRI, X-ray, etc.). Besides, in some cases, it may not be possible to diagnose or obtain information about the course of the disease on a single biomarker. Personalized diagnostic and treatment approaches are effective options. Therefore, studies on cancer biomarkers have a critical place for researchers [2,11]. Electrochemical sensor applications for biomarker detection and cancer diagnosis have always been crucial in cancer-related studies. Electrochemical sensor applications developed for this purpose and prominent studies carried out in recent years are explained in the following sections.

## 3. Application of sensors

### 3.1. Application of MIP-based sensors

**Figure 3:**
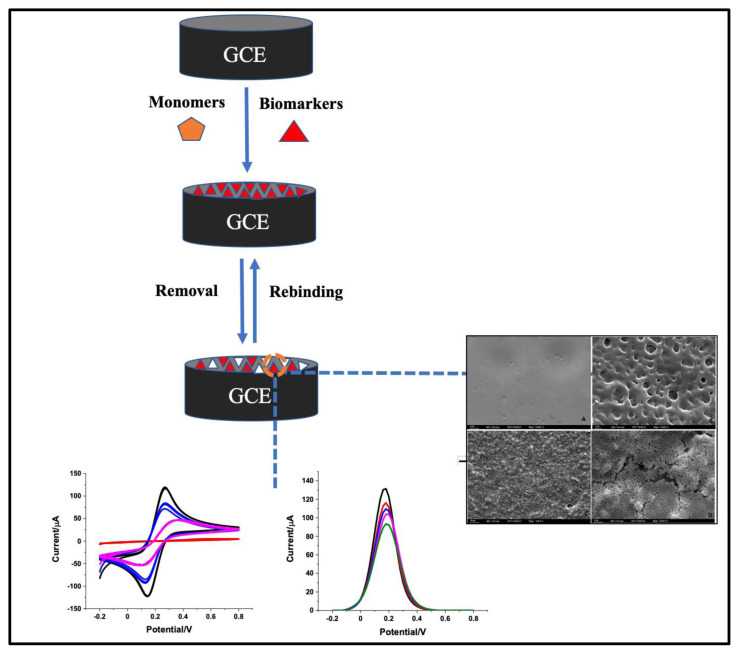
The schematic illustration of MIP-based sensor.

Molecularly imprinted polymers (MIPs), because of their superior chemical and mechanical durability, have enormous potential as synthetic recognition components in biosensors. The synthetic binding sites for MIPs are created by first allowing the polymerization around the template molecules to occur, then removing the template, according to a straightforward “lock-key” method. The molecules’ complementary structural relationships are the most crucial aspect. However, merely generating complementary sizes and shapes is frequently insufficient for selective molecular detection. Creating chemical binding sites that resemble those seen in naturally occurring receptor-target interactions is also necessary [23]. Choosing the right monomer can create high selectivity and binding sites for target molecules. However, building these receptors correctly is time-consuming because some parameters need to be optimized. Together with the target molecules’ complementary form and size, the chemical interactions that take place during the polymerization step are extremely important (Figure 3). The covalent or noncovalent interactions may be present in the prepolymerized mixture, and molecular printing techniques are called “covalent imprinting” and “noncovalent imprinting”, respectively. In “covalent imprinting”, covalent links are first formed between the template and functional monomers, and these bonds are then chemically broken during template removal.

Moreover, only reversible noncovalent interactions (such as hydrogen bonds, electrostatic interactions, and van der Waals) between the template and the functional monomer are formed in noncovalent imprinting. These noncovalent interactions may be undone by only washing the polymer with an appropriate solvent. In addition, noncovalent imprinting has the advantage that it can be performed in a variety of systems without too many limitations between the functional monomer and the template. However, in covalent imprinting, the template may be difficult to remove [24–26].

MIPs can be created for any chemical, including inorganic ions, drugs, biomarkers, nucleic acids, and proteins. They differ from natural biomolecules in several ways, including stability, specificity, ease of preparation, and miniaturization. As a result, they provide viable alternatives to the natural receptors currently used in sensor technology. However, the binding kinetics of MIPs need to be improved, analysis times need to be shortened, and the majority of the template has to be eliminated for MIPs to be successfully used in sensors. Increasing the surface-to-volume ratio and making binding sites more reachable to analytes has shown that constructing MIPs at the nanomaterials can significantly impact these problems [27,28]. The need for rapid and sensitive cancer biomarker detection is becoming more and more important recently. The efficiency of the instrument in terms of specificity, cost, detection limit, and analysis time is significantly affected by the precise design of the apparatus suitable for this use. The various detection methods for cancer biomarker detection using MIP-based sensors are summarized in Table 3.

To simultaneously detect PSA and Myo in biological samples (serum and urine), a novel dual-modality immunosensor based on MIP and a nanostructured biological sensing layer was developed by Karami et al. [9]. DSP was self-assembled on an AuSPE in the first stage. After then, the DSP-SPE was covalently linked to the target proteins. The imprinted cocktail polymer was created at the SPE surface using acrylamide as the monomer, “N,N′-methylenebisacrylamide” as the crosslinker, and PSA and Myo as the appropriate templates. The MIP-SPE was designed specifically for the impedimetric sensing of PSA and Myo. Next, using decorated magnetite nanoparticles, multiwalled carbon nanotubes (CNTs), GO, and a particular antibody for PSA, a nanocomposite (NCP) was developed (Ab). EIS, dynamic light scattering (DLS), surface plasmon resonance (SPR), and SEM were used to characterize the developed sensor and fabricated nanoparticles. The LODs were calculated 5.4 pg/mL and 0.83 ng/mL, and linear ranges were found to be 0.01–100 and 1–20,000 ng/mL for PSA and Myo, respectively. The suggested biosensor offers a significant advantage for next-generation biosensors due to its ability to determine PSA and Myo with excellent sensitivity and specificity simultaneously. Device design based on this dual analyte-specific receptors-on-chip will be important to measure a broad panel of biomarkers at incredibly low levels in the early stages of disease progression.

Zhang et al. [31] developed a MIP-based sensor based on a new signal amplification method for accurately detecting PSA. MoS_2_ and AuNPs were exchanged at the electrode surface as the sensing substrate. Surface-imprinted cavities were created using a PSA template and 4-MPBA acid monomer, and a composite made of gold polymerized methylene blue labeled with 4-MPBA was used as the tracking tag. Electrochemical characterization studies of the sensor were performed with CV and EIS measurements. The sensing substrate and tracing tag were characterized using energy dispersive spectrometer analysis, transmission electron microscopy, and SEM. After the target PSA was fixed to the sensor, an enhanced signal was generated by the coordinated electrochemical catalytic interaction of the tracking tag and the nanomaterials on the substrate. The modified sensor displayed a significant linear range from 1 × 10^−4^ to 1 × 10^4^ ng/mL; the LOD was 0.03 pg/mL. Also, such a sensor demonstrated acceptable selectivity, repeatability, and stability in the experiments, indicating an anticipated application opportunity in identifying tumor markers.

As breast cancer occurs more frequently over time, early diagnosis, patient follow-up, and treatment advice are critical. Many tumor biomarkers have been suggested to control and monitor this disease. However, CA 15-3 is currently the most significant breast cancer serum biomarker. In this study, a MIP-based electrochemical sensor developed by Pacheco et al. [40] and breast cancer detection was based on direct surface imprinting of CA 15-3 on an Au-SPE. Adsorption of CA15-3 onto the surface of the Au-SPE and electropolymerization of 2-AP around the protein adsorbed were the first two steps in the imprinting process. Hexacyanoferrate (II/III) was used as a redox probe in voltammetric analysis after the imprinted protein was extracted, which involved detecting the signals before and after protein binding. The analytical responses of imprinted and nonimprinted polymer sensors were investigated, and the proposed sensor was characterized by CV and EIS. A linear relationship was obtained between the redox probe’s peak current density and the CA 15-3 concentration logarithm between 5.0 and 500 U/mL, and the LOD was calculated as 1.5 U/mL. The developed MIP sensor enables quick analysis and is inexpensive, simple to prepare, disposable, and could be readily integrated with compact portable POC systems.

The application of a high-performance detection layer based on DMIP for the individual detection of CEA and AFP as lung cancer biomarkers were performed by Taheri et al. [43]. The antibodies of AFP and CEA on an FTO electrode were electropolymerized using PPy. Morphological and electrochemical characterization of the detection layer was performed using SEM, CV, and EIS. Methyl orange (MO) improved the conductivity of PPy and obtained the formation of MO-doped rectangular PPy nanotubes. The rebinding of template antigens was measured by impedimetric detection, and the charge transfer resistance increased as the concentrations of AFP and CEA increased. The detection limits of 1.6 pg mL^−1^ and 3.3 pg mL^−1^ and linear dynamic ranges 5.0–1 × 10^4^ pg/mL and 10–1 × 10^4^ pg/mL were found for CEA and AFP, respectively. The high sensitivity and excellent stability of the DMIP sensor made it a potential sensor for detecting AFP and CEA in serum samples, which led to satisfactory results in measuring AFP and CEA in human serum samples.

### 3.2. Application of immunosensors

**Figure 4:**
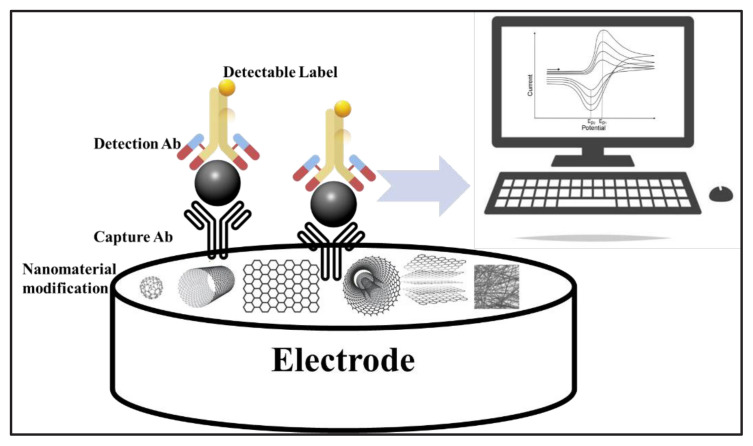
The schematic illustration of an electrochemical immunosensor.

Immunosensors are highly specific biosensors with excellent selectivity based on antigen-antibody interaction. Antibody immobilization is a critical parameter for the fabrication of immunosensor. Antigens and antibodies can be used as biological components to determine each other. The immobilization level can be increased by modifying the electrode surfaces to be used with surfactants, ionic liquids (IL), SAM (Self Assembled Monolayer) molecules, or nanoparticles with appropriate modification methods (Figure 4).

Electrochemical immunosensors have been constructed either using electroactive markers or by enzyme labeling. The immunosensor showed good sensitivity and selectivity for cancer biomarkers. The various detection methods for cancer biomarkers using immunosensor are summarized in Table 4.

Kongkaew S. et al. [48] report that the multi-electrode array (MEA) was fabricated in the presence of working, reference, and auxiliary electrodes. Prussian blue nanocubes (PBNCs) were synthesized and applied by cyclic voltammetry. Cryogel was formed with gluteraldehit dissolved in chitosan. This solution was dropped on the electrode array. The antibody immobilized the activated MEA by interacting with their amine and aldehyde groups. Then the electrode surface was blocked by 1% BSA. The four different antigens were incubated, and the analytical responses were evaluated by cyclic voltammetry. Moreover, the reduction peak current (ΔI) obtained before and after antigen incubation was calculated, and the calibration curve was drawn versus the antigen concentration. The scan electron microscope (SEM), Energy-dispersive X-ray emission spectroscopy (EDX), and atomic force microscopy (AFM) were used to characterize of MEA surface. Four cancer biomarkers (CEA, CA125, CA153, AMD CA19-9) were determined in human serum samples to detect breast cancer. The calibration curves of CEA, CA125, CA153, and CA19-9 were linear from 1.0 to 9.0 pg/mL, 0.5–15.0 mU/mL, 0.5–12.0 mU/mL, and 0.5–12.0 mU/mL, respectively. Finally, good recoveries were found, and the RSD% values were obtained low.

Yun R.Y. et al. fabricated the sandwich immunosensor to determine PHB2 in white blood cell lysates [22]. The Au electrode affinity to thiol-modified surface protein A(SH-SpA) was formed. Firstly, capture Ab immobilized the electrode surface. Next, the mixed SAM solution was prepared with MCH: MCP in the ratio of 5 : 1 to create a tunneling barrier to mediate electron transfer between the gold electrode and the redox system. PHB2, a potential biomarker for blood cancer, was immobilized on the electrode. The dAb and HRP were immobilized, respectively. The electrochemical signal amplification Horseradish peroxidase (HRP), the substrate H_2_O_2_, and the mediator hydroquinone (HQ) were used to determine PHB2 using SWV. The developed sensor was applied to WBCs lysate with healthy individuals and cancer patients. The calibration curve was drawn linearly between 0 ng/mL and 50 ng/mL with a LOD of 0.57 ng/mL. The developed sensor was compared with ELISA.

## 4. Conclusion and future perspectives

There is an increasing interest and progress in analytical applications of cancer detection. Variations of target biomarkers, improvements in sensor strategies, and changes in the diagnosis and treatment approaches have led to increased studies on sensors for cancer detection. This study explains an overview of the most significant sensor applications, MIP-based sensors, and immunosensors. The most recent studies are summarized in terms of target biomarker, sensor, technique, linear range, LOD, real sample, and recovery %. Thanks to the great advantages of immunosensors, such as very high selectivity and sensitivity due to strong antibody-antigen interaction and options of label-free and label-based analysis, they are widely used in electrochemical sensor applications. Since the antigen-antibody interaction is fundamental in immunosensor applications, antigen-based biomarkers, such as CEA, CA-125, CA19-9, and PSA, were mainly preferred as target analytes in studies conducted with this method. DPV and EIS stand out as the primarily used electrochemical techniques, while human serum samples are used as the real sample for the most part.

MIP-based sensors, on the other hand, provide a specific recognition similar to that of immunosensors with artificial components. Although the selection of materials that will create complementary structures with the target analyte and the optimization of MIP components is challenging, the high selectivity, ease of preparation, high stability, and low cost of MIP technology have the potential to be an excellent alternative to natural receptors. As in immunosensor applications, we see that CEA and PSA are often preferred as analytes in MIP-based sensor studies. In addition, it can be said that serum samples are generally applied, and DPV and SWV are prominent among electrochemical techniques.

When evaluated in terms of the future evolution of sensor studies for cancer detection, early diagnosis, and easy application purposes come to the fore. Therefore, as the first step, achieving high selectivity and reliability regarding early diagnosis is vital. Besides, laboratory-based analysis requires long processes and is expected to be replaced by lab-on-a-chip applications and portable sensors. Furthermore, decreasing the fabrication costs and assessing novel biosensor application approaches is another challenge for researchers. Considering all these, it can be foreseen that studies on the use of immunosensors and MIP-based electrochemical sensors in the field of cancer will increasingly continue since it is a versatile and open field for development.

## Figures and Tables

**Table 1:** The comparison of principles, advantages, and disadvantages of MIP-based sensors and immunosensors.

	MIP-based sensor	Immunosensor
**Principle of sensors**	Molecular recognition with specific recognition sites specific to the target molecule.	Antibody-antigen interactions
**Advantage**	Good selectivity and sensitivity High porosity Low cost Lower LOD and LOQ values Easy preparation Less time consuming High mechanical stability High thermal stability The high surface density of polymer chains High stability of the coated layer Great specific recognition sites	High specificity and robust Real-time analyses Fast detection Insensitivity to environmental condition changes No sample pretreatment The application for the detection of a wide range of analytes
**Disadvantage**	Poor reproducibility Relatively long response time Narrow linear range Deterioration of cavities after removal Requirement of the high amount of porogen structures Damage of cavity sites during analysis	Low sensitvity High cost Short lifetime Low levels of stability complexity Changing the biomolecule’s activity Interference from contaminants

**Table 2:** Classification of prominent cancer biomarkers.

Biomarker	Structure	Source	Cancer Type	Ref.
CEA	Circulating carcinoma protein	Invasive, blood	Lung and breast	[2],[6]
Prostate-specific antigen (PSA), free PSA (fPSA), prostatic acid phosphatase (PAP), myoglobin (Myo)	Circulating carcinoma protein, enzyme	Invasive, blood	Prostate	[2],[7]–[9]
CA 19-9, CA 125	Protein-based	Invasive, blood	Pancreatic, colon, breast, ovarian	[2],[6],[10]
p53	Nucleic acid-based	Invasive, blood	Lung, colorectal, pancreatic, ovarian	[2],[8],[10]
Vascular endothelial growth factor (VEGF)	Protein-based	Invasive, blood	Lung, brain, gastrointestinal, urinary tract, breast, stomach	[6],[10],[11]
Alpha-fetoprotein (AFP)	Protein-based	Invasive, blood	Lung, pancreatic, liver	[6],[7]
microRNA (miRNA)	Genetic	Noninvasive, saliva	Ovarian, lung, breast, pancreatic	[2],[6]
Ethanol	Alcohol	Noninvasive, breath	Lung, liver, colorectal	[2],[6]
Circulating tumor cells (CTCs)	Cell	Invasive, blood	Breast, colorectal, prostate, pancreatic, hepatic, pulmonary	[2],[12],[13]
Human epidermal growth factor receptor 2 (HER2)	Circulating carcinoma protein, genetic	Invasive, blood	Breast, stomach	[2],[10],[11]
Estrogen receptor (ER), progesterone receptor (PR), Mucin1 (MUC1), CA15-3, Breast cancer antigen 1/2 (BRCA1/BRCA2), inhibitor of growth protein 1 (ING-1), calreticulin (CALR)	Genetic, protein	Invasive, blood	Breast	[8],[11],[14],[15]
Panel of Kirsten rat sarcoma viral oncogene homolog (KRAS), proto-oncogene B-Raf and v-Raf murine sarcoma viral oncogene homolog B (BRAF), Matrix metallopeptidase 9 (MMP9), melanotransferrin (TRFM), Retinol Binding Protein 4 (RBP4), Trefoil Factor 3 (TFF3), Claudin7 (CLD7)	Miscellaneous	Invasive, blood	Colorectal	[11],[16]
E-cadherin, Fibroblast growth factor receptors (FGFR), c-MET, alcohol dehydrogenases (ADH), MMP9, Transforming Growth Factor Beta 1 (TGFB1), sex-determining region Y box 9 (SOX9), Interleukin-6 (IL-6)	Miscellaneous	Invasive, blood	Stomach	[11]
CYFRA 21-1 (Cytokeratin 19)	Protein-based	Invasive, blood	Lung, bladder	[8],[10]
Annexin A2 (ANXA2), serum amyloid A1 (SAA1), squamous cell carcinoma antigen (SCC)	Protein-based	Invasive, blood	Lung	[8],[10]
Fibrin/fibrinogen degradation products (FDP), Nuclear Matrix Protein Number 22 (NMP22), hyaluronic acid (HA) and hyaluronidase (HAse), Bladder Cancer-Specific Nuclear Matrix Proteins-4 (BLCA4)	Miscellaneous	Invasive, blood	Bladder	[8]
Tyrosinase, NY-ESO-1	Miscellaneous	Invasive, blood	Melanoma	[8]
Anti-alpha-fetoprotein (AFP), Human chorionic gonadotropin (β-hCG)	Miscellaneous	Invasive, blood	Testicular	[8]
Galectin-3	Protein-based	Invasive, blood	Breast, gastrointestinal, lung, or ovarian cancer, melanoma, non-Hodgkin’s lymphoma	[17]
Apolipoprotein-A1 (Apo-A1)	High-density lipoprotein	Noninvasive, urine	Bladder	[18]
Engrailed 2 (EN2)	Genetic	Noninvasive, urine	Prostate	[19]
Human epididymis protein 4 (HE4)	Protein-based	Invasive, blood	Ovarian	[20]
Cluster of differentiation 147 (CD147)	Exosomal biological molecule	Invasive, blood	Colorectal, nonsmall-cell lung	[21]
Prohibitin 2 (PHB2)	Protein-based	Invasive, blood	Liver, colon, breast, prostate, blood	[22]

**Table 3:** MIP-based sensor applications for selected cancer biomarkers.

Analyte	Sensor	Technique	Linear Range	LOD	Real Sample	Recovery %	Ref.
PSA	Aptamer–AuE/MIP	EIS	100 pg/mL–100 ng/mL	1.0 pg/mL	NR	NR	[29]
PSA	Py-EP-GCE/MIP	DPV	0.89–10.93 ng/mL	2.0 pg/mL	Blood serum	NR	[28]
PSA Myo	DS-SPE/MIP	EIS	0.01–100 ng/mL 1.0–20,000 ng/mL	5.4 pg/mL 0.83 pg/mL	Serum Urine	98.17–102.89 98.73–102.87	[9]
PSA	PPy-SCE/MIP	SWV	30–300 ng/mL	30 ng/mL	Serum	NR	[30]
PSA	AuNPs/MoS2/4-MPBA/GCE/MIP	DPV	1 x 10^−^^4^–1 × 104 ng/mL	0.3 pg/mL	Serum	97.0–103.0	[31]
PSA	PTB/GA-Cys A/MIP	DPV	1–60 μg/L	1.0 μg/L	Serum	NR	[32]
CEA	Ag/PPy/SPE/MIP	CV DPV EIS	0.05–1.25 pg/mL	1.25 pg/mL	Urine	NR	[33]
CEA	AuNPs/PTh/DA/GCE/MIP	DPV	0.001–1000 ng/mL	0.2589 pg/mL	Serum	88.7 – 124.6	[34] [34]
CEA	PAP/hCCl/APBA/FTO/MIP	EIS	1.5 μg/L–2.5 ng/mL	3.0 ng/mL	Fetal bovine serum	NR	[35]
CEA	GO/Chitosan/Bio-ePADs/MIP	DPV	1.0–1000 ng/mL	0.32 ng/mL	Serum	NR	[36]
AFP	[(Cys)VIMBF4]/AuNPs/GCE/MIP	DPV	0.03–5.0 ng/mL	2.0 pg/mL	NR	NR	[37]
CA-125	GNEE/MIP	DPV	0.5–400 U/mL	0.5 U/mL	Blood serum	NR	[38]
CA-125	Py/AuE/MIP	SWV	0.01–500 U/mL	0.01 U/mL	Serum	91.0–105.0	[39]
CA15-3	2-AP/AuSPE/MIP	DPV	5.0–500 U/mL	1.5 U/mL	Serum	72.82–87.0	[40]
CA15-3	PTB/AOT/AuSPE/MIP	DPV	0.1–100 U/mL	0.1 U/mL	Serum	NR	[41]
CA15-3	CNE/AuNPs/MIP	CA	5.0–35 U/mL	1.16 U/mL	Serum Saliva	101.8–104.3 61.7–75.8	[42]
AFP CEA	PPy/FTO/DMIP	EIS	5.0–1 × 10^4^ pg/mL 10.0–1 × 10^4^ pg/mL	1.6 pg/mL 3.3 pg/mL	Serum	96.0–98.8	[43]
EGFR	AuSPE/MIP	DPV	10–70 ng/mL	1.6 ng/mL	Serum	80.6–90.2	[44]
Galectin-3	AP/SPE/MIP	DPV	0.5–5000 ng/mL	0.5 ng/mL	Serum	NR	[17]
Cyt c	o-PD/AuE/MIP	DPV	0–7 pg/mL	4.2 x 10^−^^2^ pg/mL	Serum	80.0–98.0	[45]
HER2	EDOT/AuNS/LSGE/MIP	SWV	1.0–200 ng/mL	0.43 ng/mL	Serum	109.5–112.0	[46]

NR: Not reported, PSA: Prostate specific antigen, Myo: Myoglobin, CEA: Carcinoembryonic antigen, AFP: Alpha fetoprotein, CA: Cancer antigen, EGFR: Epidermal growth factor receptor, VEGF: vascular endothelial growth factor, Cyt c: Cytochrome c, HER2: Human epidermal growth factor receptor 2, EIS:Electrochemical Impedance Spectroscopy, CA: Chronoamperometry, EP: Electropolymerization, DSP: 3,3′-dithio-dipropionic acid di(N-hydroxysuccinimide ester), DPV: Differential Pulse Voltammetry, AuE: Gold electrode, GCE: Glassy carbon electrode, SPE: Screen printed electrode, SCE: Saturated calomel electrode, FTO: Fluorine doped tin oxide, GNEE:Gold nanoelectrode ensemble, SWV: Square wave voltammetry, AuSPE: Screen-printed gold electrode, LSGE: Laser scribed graphene electrode, Py: Pyrrole, AuNPs: Gold nanoparticles, MoS_2_: Molybdenum disulfide, CV: Cyclic Voltammetry, 4-MPBA: 4-mercaptophenylboronic acid, PTB: poly (Toluidine Blue), GA-Cys A: Glutaraldehyde-cysteamine, PPy: Polypyrrole, Ag: Silver tracks, DA: Dopamine, PTh: Polythionine, PAP: Polyaminophenol, hCCl: Homemade carbon ink, APBA: Aminophenylboronic acid, Bio-ePADs: Movable valve paper-based device, GO: Graphene oxide, [(Cys)VIMBF4]: 1-[3-(N-cystamine)propyl]-3-vinylimidazolium tetrafluoroborate ionic liquid, 2-AP: 2 -aminophenol, AOT: 8-amino-1-octa-nethiol, CNE: Carbon nanotube electrode, DMIP: Dual-template molecularly imprinted polymer, o-PD: orto-phenylenediamine, EDOT: 3, 4-ethylenedioxythiophene, AuNS: Gold nanostructures

**Table 4:** Immunosensor applications for selected cancer biomarkers.

Analyte	Sensor	Technique	Linear Range	LOD	Recovery	Real Sample	Ref
HER2	Au@PdAg DBNRs/GCE	DPV	0.001–100 ng/mL	0.25 pg/mL	100.2–104.5	Serum sample	[47]
CEA	Cryogel-PBNCs/ME/GCE	CV	0.001–0.009 ng/mL	0.79 ng/mL	97–100	Human serum sample	[48]
CA125			0.0005–0.015 U/mL	0.37 U/mL	95.1–104		
CA153			0.0005–0.012 U/mL	0.49 U/mL	97–102		
CA19-9			0.0005–0.012 U/mL	0.48 U/mL	99–100.8		
CYFRA 21-1	AuNPs/P(PyAmn)/ITO	EIS	0.015–90 pg/mL	4.59 pg/mL	95.5–106.7	Serum sample	[49]
CA125	AuNPs@MWCNTs/GCE	SWV	0.0004–4 U/mL	0.0004 U/mL	NR	NR	[50]
IL-6	Au/CF composite electrodes	DPV	1 fg/mL–1 μg/mL	0.056 fg/mL	NR	Serum sample	[51]
CA125	Eu MOF@Isolu−Au NPs/GCE	Electro chemiluminescence	0.005–500 ng/mL	0.37 pg/mL	92.6–113.9	Serum sample	[52]
HE4				1.58 pg/mL	101.6–115.8		
Cyfra-21-1	Ab_2_-Fc/Ag/BSA/Ab1/MB/CdTe/MoS_2_/GCE	DPV	10 pg/mL–10000 ng/mL	10 pg/mL	100–104	Serum sample	[53]
Thyroglobulin	Ab1-CD-CNTs/GCE	DPV	2–200 ng/mL	0.5 ng/mL	96.6–99.2	Serum sample	[54]
CEA	NHMN/Au/GCE	DPV	0.005–10.0 ng/mL	0.6 ng/mL	95.3–107.6	Serum sample	[55]
Apo-A1	BSA/Ab1/Chit/MoS2/GQD/GCE	EIS	1.00 pg/mL–1.00 μg/mL	0.30 pg/mL	96.4–109.1	Serum sample Urine sample	[18]
CALR	rGO-PPyNH2/ITO	EIS	0.025–75 pg/mL	10.4 fg/mL	86.0–107.3	Serum sample	[15]
Claudin7	Nanosized MIL-125-NH_2_ particles microfluidics sensor	Amperometry	2–1000 pg/mL	0.1 pg/mL	NR	Colon cancer cell line	[16]
AFP	Ni-Co MOF/GNP electrode	DPV	1–200 ng/mL	0.3 ng/mL	90.0–105.9	Serum sample	[56]
HE4	PtNi NCAs/GCE	DPV	0.01–100 ng/mL	0.11 pg/mL	99.9–101.3	Serum sample	[57]
CEA	SLB-WS2@ MWCNT/GCE	EIS	1x10^−^^7^–1000 ng/mL	0.2 pg/mL	98–102	Serum sample	[58]
CEA	Au@NBOF NSs/GCE	EIS	100 fg/mL–200 ng/mL	9.57 fg/mL	99–102.7	Serum sample	[59]
EN2	PABA film/Au electrode	SWV	10^−^^5^ ng/mL–1 μg/mL	10^−^^5^ ng/mL	NR	NR	[19]
PG I	PANI/MoS_2_@Cu_3_Pt NPs/SPE	DPV	500 pg/mL–400 ng/mL	167 pg/mL	NR	Serum sample	[60]
He4	BSA/Ab/PAH/BPNS/GCE	DPV	0.1–300 ng/mL	0.1 ng/mL	NR	Serum sample	[20]
SCCA	PtCo BNCs/GCE	DPV	0.001–120 ng/mL	0.33 pg/mL	98.5–110.0	Serum sample	[61]
NSE	AuNPs microelectrode	DPV	1–750 ng/mL	0.34 ng/mL	NR	Serum sample Saliva sample	[62]
SCCA	PtFe/H-NCFs/GCE	DPV	0.01 pg/mL–10 ng/mL	0.003 pg/mL	96.0–100.0	Serum sample	[63]
CEA	ZrO2-rGO-IL/GCE	DPV	100.0 fg/mL–5.0 ng/mL	2.25 fg/mL	>94	Serum sample	[64]
CA19-9	PDA/MB/GO-CNT/ITO	DPV	0.1 mU/mL–100 U/mL	0.54 nU/mL	95.0–110	serum sample	[65]
CA125	MOF/COF/CNT/CPE	DPV	0.0001–100 U/mL	0.000088 U/mL	96.1–109.5	Serum sample	[66]
MCM5	11-MAU/Gold electrode	EIS	10^−^^6^ –10^−^^11^ g/mL	2.9 10^−^^11^ g/mL	NR	NR	[67]
PSA	MB/PS	Amperometry	10–1500 pg/mL	2 pg/mL	95	Serum sample	[68]
CALR	SWCNTs-PPepx/ITO Electrode	EIS	0.015–60 pg/mL	4.6 fg/mL	95.3–108.3	Serum sample	[69]
CD147	MBs/SPCE	Amperometry	0.096–5.0 ng/mL	29 pg/mL	NR	Serum sample	[21]
CEA	Cu-TCPP-PB/GCE	DPV	0.1–160 ng/mL	0.03 ng/mL	NR	Serum sample	[70]
CA125			0.5–200 U/mL	0.05 ng/mL	NR		
CA 242	PdAgPt/MoS2/GCE	CA	1 × 10^−4^ U/mL –1 × 10^2^ U/mL	3.43 × 10^−5^ U/mL	98–107	Serum sample	[71]
IL-8	Aminothiol/MWCNTs/Ab/Au Electrode	EIS	1–1000 pg/mL	0.1 pg/mL	NR	Serum sample	[72]
CEA	MoS2/Cs/Au/Anti-CEA/CEA/PGE	DPV	0.01–10 ng/mL	1.93 ng/mL	98	Serum sample	[73]
pro-SFTPB	Ag/BSA/Ab1/AuNPs/BP nanosheets/GCE	Amperometriy	10 pg/mL–100 ng/mL	5.3 pg/mL	97.8–101.6	Serum sample	[74]
CA19-9	BSA/anti-CA19-9/MPA/ME/Au	DPV	0.05–500 U/mL	0.01 U/mL	102.4–115.0	Serum sample	[75]
AFP	Fe_3_O_4_NPs@COF/AuNPs/GCE	SWV	0.01–1 pg/mL	3.30 fg/mL	100	Serum sample	[76]
SP17	BSA/anti-SP17/APTMS/ITO	DPV	100–5000 pg/mL	70.07 pg/mL	NR	Serum Sample	[77]
β-1,4-GalT-V	MCH/11-MUA/SPAuE	EIS	0.23–722 ng/mL	0.32 ng/mL	83.8 – 100	Serum Sample	[78]
PSMA	Nanofiber electrode	EIS	10–200 ng/mL	9.5 ng/mL	NR	NR	[79]
PSMA	Cys-AuNP/SPGE	DPV	0–5 ng/mL 5–250 ng/mL	48.2 ng/mL	>90	PSMA–expressing PCa cells	[80]
			0–100 cells/mL 100–400 cells/mL	5 cells/mL 22 cells/mL		LNCaP cell	
HER2	GCE/CoP-BNF/SNGQDs@AuNPs/Trasmatuzab	EIS	1–7 ng/mL	0.0327 ng/mL	80–110	Serum Sample	[81]
PHB2	MCH:MCP/Au electrode	SWV	1.56–50 ng/mL	0.63 ng/mL	89.1–104.7	White blood cell lysate	[22]
NSE	CoFe_2_O_4_@Ag/Ab2/anti-NSE/NSE/BSA / Ab1/anti-NSE/MoS2@AuNPs/rGO/GCE	SWV	0.01–1.00 pg/mL	3.0 fg/mL	99.8 – 100.5	Serum sample	[82]
MMP-2	Au/PEI/Ab/BSA/Au disc electrode	SWV	2.0 pg/mL–5.0 μg/mL	10 fg/mL	96.8 – 101.9	Rat plasma	[83]
AFP	BSA/anti-AFP/CS-AuNPs/GO/GCE	DPV	0.1–100 ng/mL	0.041 ng/mL	NR	NR	[84]
CD-44	GO-IL-AuNPs/GCE	DPV	5.0 fg/mL–50.0 μg/mL	2.0 fg/mL	NR	Serum sample	[85]
		EIS		1.90 fg/mL			
PSA	P(CS)-AgNPs-WSN-Ab1-BSA-PSA-Ab2/GCE	DPV	0.002–60 μg/L	0.002 μg/L	NR	Plasma sample	[86]
HER-2	AuNP-Ab-HRP	CA	0–75 ng/mL	30 pg/mL	100.0	Serum sample	[87]
HER-1							
CA125	DSPCE/RGO/PTH/AuNP	DPV	1–100 pg/mL 1–50 ng/mL	0.069 pg/mL 1.640 ng/mL	94.8–104.5	Serum sample	[88]
		SWV	1–100 pg/mL 10–50 ng/mL	0.066 pg/mL 1.470 ng/mL			
HE4		DPV	1–100 pg/mL 1–50 ng/mL	0.058 pg/mL 1.32 ng/mL			
		SWV	1–100 pg/mL 1–50 ng/mL	0.164 pg/mL 2.22 ng/mL			
CA19-9	3D cactus-like NiCo-LDH/CuSe/CC sensor	DPV	0.001–100 U/mL	0.0005 U/mL	99.0–100.3	Serum sample	[89]
PD-L1	CAbPD-L1-MB/SPCE	Amperometry	240–5000 pg/mL	86 pg/mL	NR	Cancer cell lysate	[90]
HIF-1α	CAbHIF-1α-MB/SPCE		930–10,000 pg/mL	279 pg/mL			

NR: not reported, HER2: human epidermal growth factor receptor 2, Au@PdAg DBNRs:Au@PdAg dog-bone-like nanorods, Cryogel-PBNCs/MEA: cryogel-Prussian blue nanocubes modified multielectrode, (AuNPs/P(PyAmn)) array: gold nanoparticles and amino-substituted-pyrrole polymer, IL-6: interleukin-6, Au/CF: Au-integrated flexible carbon fiber, Eu MOF@Isolu−Au NPs: Eu metal−organic framework-loaded isoluminol−Au nanoparticles, MB/CdTe/MoS2:methylene blue/cadmium telluride/molybdic sulfide, GCE: glassy carbon electrode, Ab2-Fc: amino-functionalized secondary antibodies through amido bond, CD-CNTs: β-cyclodextrinfunctionalized carbon nanotubes nanohybrid, NHMN/Au: N-doped hollow mesoporous nanocarbon spheres/gold hybrids, MoS2/GQD: molybdenum disulfide/graphene quantum dot, CHIT: Chitosan, rGO-PPyNH2: reduced graphene oxide/amino substituted polypyrrole polymer, Ni-Co MOF: Ni-Co metal-organic framework, QD: quantum dots, NCAs: PtNi nanocubes assemblies, SLB-WS2@MWCNT: SLB is tethered on stable tungsten disulfide decorated MWCNT, Au@NBOF NSs: Au NPs inside the mesoporous NBiOF nanospheres, PABA: poly para amino benzoic acid, MoS2 NFs: rhombohedral Cu3Pt and MoS2 nanoflowers, HE4: human epididymis protein 4, BPNS: black phosphorus nanosheets, PAH: poly(allylamine hydrochloride), SCCA: squamous cell carcinoma antigen, PtCo BNCs: PtCo highly branched nanocrystals, PtFe/H-NCFs: N-doped carbon nanoflowers, PDA: polydopamine, MB: methylene blue, GO-CNT: graphene oxide-carbon nanotubes, ITO: indium tin oxide, MOF: metal organic framework, CNT: carbon nanotube, CPE: carbon paste electrode, 11-MUA: 11-mercaptoundecanoic acid, PS: polystyrene sheets, MB: magnetic beads, CALR: calreticulin, hTERT: human telomerase reverse transcriptase, CEA: carcinoembryonic antigen, CA125: carbohydrate antigen 125, PB: prussian blue, MoS2: molybdenum disulfide, IL-8: interleukin-8, PGE: pencil graphite electrodes, PSA: prostate-specific antigen, WPN: wrinkled silicate nanoparticles, pro-SFTPB: Pro-surfactant protein B, BP nanosheets: few-layer black phosphorous, BSA: Bovine Serum Albumin, MPA: 3-mercaptopropionic acid ME: β-mercaptoethanol, COF: covalent organic framework, SP17: Sperm protein 17, β-1,4-GalT-V: β-1,4-Galactosyltransferase-V, MCH: 6-Mercapto-1-hexanol, PSMA: prostate-specific membrane antigen, CoP-BNF: porphyrin binuclear framework, PHB2: prohibitin 2, NSE: neuron-specific enolase, AuNPs@MoS2/rGO: molybdenum disulfide and reduced graphene oxide, MMP-2: matrix metalloproteinase-2, AFP: Alpha-fetoprotein, CS-AuNPs: Chitosan-modified gold nanoparticles, CD44:cluster of differentiation-44, HIF-1α: hypoxia-inducible factor 1 alpha, GNP: graphene nanosheet-printed polyethylene terephthalate, Apo-A1: apolipoprotein-A1, EN2: Engrailed 2, APTMS: (3-Aminopropyl) trimethoxysilane, SNGQDs: sulfur and nitrogen doped graphene quantum dots, ECL: Electrochemiluminescence, AuNP: gold nanoparticles, PTH: Polythionine, DSPCE:Dual Screen Printed Electrode, NiCo-LDH: nickel cobalt layered double hydroxide, CC: directly hydrothermally grown on carbon cloth

## Data Availability

Not applicable.
